# Examining Risk Factors in the Cannabis–Suicide Link: Considering Trauma and Impulsivity among University Students

**DOI:** 10.3390/ijerph19159307

**Published:** 2022-07-29

**Authors:** Ayeila Z. B. Daneshmend, Jayme Stewart, Dana A. Jarkas, Sabina I. Franklyn, Robert L. Gabrys, Zachary R. Patterson, Alfonso Abizaid, Kim G. C. Hellemans, Robyn J. McQuaid

**Affiliations:** 1 Department of Neuroscience, Carleton University, Ottawa, ON K1S 5B6, Canada; danajarkas@cmail.carleton.ca (D.A.J.); robgabrys@gmail.com (R.L.G.); zacharypatterson@cmail.carleton.ca (Z.R.P.); alfonsoabizaidbucio@cunet.carleton.ca (A.A.); kimhellemans@cunet.carleton.ca (K.G.C.H.); robynmcquaid@cunet.carleton.ca (R.J.M.); 2 University of Ottawa Institute of Mental Health Research at the Royal, Ottawa, ON K1Z 7K4, Canada; 3 Department of Psychology, Carleton University, Ottawa, ON KIS 5B6, Canada; jaymestewart@cmail.carleton.ca (J.S.); sabinafranklyn@cmail.carleton.ca (S.I.F.)

**Keywords:** cannabis, suicide, trauma, impulsivity, stress

## Abstract

Cannabis is a commonly used substance among university students that may have several negative health repercussions, including suicidal ideation (SI) and suicide attempts (SA). The factors that contribute to or help explain this relation remain uncertain. Earlier negative experiences, especially trauma encountered during early life, have been associated with the development of psychopathology upon later stressor encounters. In the current study, we examined the associations between SI and SA with problematic cannabis use among young adults and the role of earlier trauma experiences and trait impulsiveness in understanding this link. Among university students (N = 539), problematic cannabis use was moderately related to lifetime and past-12-months suicidal ideation and attempts. Impulsiveness mediated the relationship between problematic cannabis use and lifetime SI and SA. Moreover, previous life trauma moderated the relationship between problematic cannabis use and SA, such that the association between problematic cannabis use and SA was stronger among those who experienced high levels of trauma. These findings highlight behavioral and environmental factors that could predict suicide ideation and attempts among young cannabis users. Accordingly, trait impulsiveness and early trauma experiences should be considered, alongside problematic cannabis use, in suicide-risk detection and prevention strategies among young adults.

## 1. Introduction

Suicide is a global health concern, with approximately 800,000 individuals dying by suicide annually [1,2]. Strikingly, suicide is the second-leading cause of death among young adults, only exceeded by accidents [1,2]. Psychosocial risk factors, including elevated levels of stress, loneliness, and lack of social support; serious and/or current mental illness; family history of suicidality; and psychological traits, such as impulsiveness and experiences of trauma [3], predict suicidal thoughts and behaviors [4,5] and are currently considered in suicide-prevention strategies [6]. Moreover, suicidal ideation (SI) and previous suicide attempts (SA) have been identified as potent predictors of death by suicide and are common among emerging adults, who face a unique set of stressors associated with the transition to adulthood [7,8].

Suicidal thoughts and behaviors have also been associated with recent problematic cannabis use, which has garnered increased attention in suicide research [9,10]. Cannabis is among the most commonly used psychoactive substances worldwide, and cannabis policies have shifted toward legalization in several countries, despite some of the suspected harms associated with problematic cannabis use. With recent legalization, the perceived risk associated with cannabis use is declining and is mirrored by increased cannabis consumption, particularly among emerging and young adults [11,12]. For instance, approximately 25% of undergraduate university students, a subpopulation of emerging adults, reported past-30-day cannabis use [13]. University students who used cannabis in the past 30 days reported increased perceived burdensomeness and lower levels of belongingness, which may indirectly contribute to an increased risk of SI [14]. Likewise, nearly 15% of undergraduate students who reported past-year cannabis use experienced adverse outcomes associated with cannabis use, including suicidal thoughts [15]. The dearth of literature examining the link between problematic cannabis use and suicidal thoughts and behaviors has led to inconsistent conclusions, with some studies suggesting that cannabis use specifically does not influence suicidal outcomes, while others highlighted the elevated risk [16,17,18]. Thus, further investigation into the potential role of problematic cannabis use in the etiopathogenesis of suicidal thoughts and behaviors is critical.

Numerous psychiatric and trauma-related disorders tend to co-occur with problematic cannabis use, although the majority of individuals who use cannabis will not develop a cannabis-use disorder (CUD) and/or mental illness [19,20]. Emerging evidence suggests, however, that psychological disorders such as major depressive disorder may promote problematic cannabis use and CUDs [18,21,22]. Consistent with this finding, individuals who reported suicidal thoughts and behaviors and have a pre-existing mental illness were more likely to report subsequent cannabis use [18], although several studies reported a cannabis–suicide link independent of comorbid disorders [23]. Interestingly, one investigation found no association between problematic cannabis use and suicidal behaviors among individuals with an existing mental illness, highlighting the importance of studying this relationship in both psychiatric and general populations [24]. Relative to pre-existing psychopathological and heritable traits, individuals who used cannabis problematically were 2.5 to 2.9 times more likely to report SI and SA compared to their co-twin without a cannabis dependence [25]. Expanding upon these findings, a subsequent investigation observed an increased risk of suicidal ideation among monozygotic twins who used cannabis frequently (OR = 2.47, 95% CI [1.19–5.10]), in comparison with their identical co-twin with less frequent use [21]. Moreover, distinct associations between problematic cannabis use and suicidal thoughts and behaviors suggest that cannabis may influence suicidality via pathways independent of pre-existing vulnerabilities [7,26,27].

The prospective pathways driving the link between suicidal outcomes and problematic cannabis use prompts further investigation of the psychosocial and experiential determinants that may both mediate and moderate the relationship between problematic cannabis use and SI and SA. Gender, ethnicity, age of initiation, frequency and heaviness of cannabis use, emotional regulation, trait impulsivity, and trauma before the age of 18 have been suggested as potential risk factors for cannabis-associated suicidal thoughts and behaviors [9,28,29]. Moreover, symptoms of cannabis dependence have been associated with an elevated incidence of impulsive, unplanned suicide attempts [26,28,30]. Impulsivity is a heterogeneous construct with diverse underpinnings thought to differentially confer the risk of suicidal thoughts and behaviors [31]. While some studies indicated that impulsivity is not directly associated with problematic cannabis use [30], others suggested that impulsivity may instead be a distal factor in the cannabis–suicide relationship, contributing to the development of suicidal ideation but not the transition from ideation to attempt [7]. Yet, evidence of exacerbated impulsivity has been consistently linked with problematic cannabis use [32] and might mediate the transition from SI to SA [33]. The processes leading to impulsivity could be attributed to several factors, including stressor-provoked neurochemical changes [34]. Additionally, increased impulsivity and the likelihood of using cannabis and experiencing suicidal thoughts and behaviors into adulthood may be due to protracted effects of traumatic events that occurred during early life or adolescence [17,35].

Several investigations have found that early life adversity is prevalent among undergraduate university students [36,37], yet how this impacts suicidality in relation to problematic cannabis use remains unknown. It is well-established that individuals who experienced childhood trauma (abuse) are at elevated risk of mental disorders and suicide attempts [38]. Furthermore, several investigations have observed that individual subtypes of childhood trauma including emotional, physical, and sexual abuse were associated with two- to three-fold increased risk of suicide attempts, with similar findings for suicide ideation [39]. Thus, childhood trauma could act as a modifier in the relationship between cannabis use and SI and SA, effectively acting as a risk factor for trait impulsivity and problematic cannabis use, contributing to the development of SI and SA.

### The Current Research

Given the potential links between problematic cannabis use, trait impulsivity, and earlier traumatic experiences, the present study investigates the cannabis–suicide link among a university-student sample with a focus on, first, elucidating whether problematic cannabis use is associated with SI and SA; then, examining if impulsive traits mediate the relationship between problematic cannabis use and suicidality; and, lastly, determining if previous trauma experiences play a moderating role in the problematic cannabis use and SI and SA relationship. It was hypothesized that among young adults:

(1) Problematic cannabis use would be associated with increased reports of suicidal ideation and suicide attempt.
(2) Impulsive traits would mediate the relationship between higher problematic cannabis use and greater endorsement of suicidal thoughts and behaviors.
(3) Trauma experiences before the age of 18 would moderate the relationship between cannabis use and all suicide outcomes assessed, such that heavier cannabis use would predict greater endorsements of suicide ideation and attempts, especially among those with higher trauma scores (flow diagrams can be found in Supplementary Materials, Figures S1–S3).

## 2. Methods

### 2.1. Participants

Participants included 539 undergraduate students *(M*_age_ = 19.38, *SD*_age_ = 2.15, age range = 17–29) recruited from a Canadian university. The sample predominantly identified as women (76.3%; *n =* 411), followed by men (23.2%; *n* = 125) and gender non-conforming (0.6%; *n* = 3). Participants self-reported their ethnicity as White/European (59.5%; *n* = 320), Black (11%; *n* = 59), Arab/West Asian (7.6%; *n* = 41), Asian (6.1%; *n* = 33), South Asian (5.8%; *n* = 31), Latin American/Hispanic (2.6%, *n* = 14), South-East Asian (1.9%, *n* = 10), Indigenous (0.6%, *n* = 3), and Other/Mixed Ethnicities (10.1%, *n* = 27).

### 2.2. Procedure

Participants were recruited from the university’s online computerized research platform to participate in a laboratory-based study in exchange for course credit. Inclusion criteria included being 17–29 years of age and English-speaking. Participants provided informed consent followed by completing questionnaires assessing demographic information, problematic cannabis use, childhood trauma, impulsiveness, and suicidal thoughts and behaviors (i.e., suicidal ideation and suicide attempt). Upon completion, participants were debriefed on the rationale and purpose of the investigation and provided with course credit for courses associated with the university’s online computerized research platform. This project was part of a larger biological study focused on examining transdiagnostic symptom profiles of depression and anxiety [40]. The research received approval from the Research Ethics Boards at Carleton University and the Royal Ottawa Mental Health Centre.

### 2.3. Measures

***Problematic Cannabis Use.*** The 8-item Cannabis Use Disorder Identification Test-Revised (CUDIT-R [41]) was used to measure recent problematic cannabis use within the last six months. Participants respond to questions assessing the occurrence of use (e.g., *“How often do you use marijuana?”*) and dependence *(e.g.*, *“How often in the last 6 months did you fail to do what was expected of you because of using cannabis?”*), according to a five-point Likert scale ranging from 0 to 4. This total score generated by summing all items (α = 0.86) represented problematic cannabis use and was used as a continuous variable in all statistical models. In addition to the total score, the following frequency categories were also calculated for descriptive statistics and gender differences: no use (0), non-problematic (<8), potential problematic use (8–12), and possible cannabis-use disorder (13+), in line with previous investigations [42,43].

***Impulsiveness.*** The Barratt Impulsiveness Scale-11 (BIS; [44]) is a 30-item self-report questionnaire assessing impulsiveness. Items are scored on a 4-point Likert scale, ranging from 1 (‘rarely/never’) to 4 (‘almost always’). Responses are summed to form a total score (*α* = 0.80), with three higher-order factors: attentional impulsiveness (*α* = 0.69), motor impulsiveness (*α* = 0.63), and nonplanning impulsiveness (*α* = 0.68), and six lower-order factors: attention, cognitive instability, motor, perseverance, self-control, and cognitive complexity (the present study only used BIS total and higher-order factor scores to reduce the total number of statistical analyses being conducted). The BIS is a well-validated and reliable assessment that has demonstrated strong internal consistency in previous research [45].

***Childhood Trauma.*** The Childhood Trauma Questionnaire—Short Form (CTQ; [46]) is a 25-item self-report retrospective questionnaire used to measure participants’ traumatic experience, including emotional and physical neglect as well as emotional, physical, and sexual abuse before the age of 18. Responses are scored according to a 5-point Likert scale, ranging from 1 (‘never true’) to 5 (‘very often true’). A total trauma score was created by summing all items (α = 0.90). The CTQ is a well-validated and reliable assessment and has demonstrated strong internal consistency in previous research [47].

***Suicidal Thoughts and Behaviors.*** To assess suicidal thoughts and behaviors, participants reported if they had experienced suicidal ideation over the course of their lifetime (lifetime SI) and within the past 12-months (past-12-months SI), as well as if they had attempted suicide over the course of their lifetime (lifetime SA) and within the past 12-months (past-12-months SA); responses were binary (Yes/No). These questions were a modified adaptation of two questions from the suicidality section of the Composite International Diagnostic Interview (CIDI) 3.0 [48] and were designed to meet our Review Ethic Board’s requirements. All participants had the option of refraining from answering these and all other survey questions.

### 2.4. Statistical Analysis

Data analyses were conducted using IBM SPSS Statistics, Version 27.0 (IBM Corp., Armonk, NY, USA). Data screening and cleaning was conducted to detect out-of-range scores due to human error in data entry and bring outliers (*z* = ±3.29) into range. Bivariate correlations were used to assess the relationships between suicidal thoughts and behaviors, problematic cannabis use, childhood trauma, and impulsiveness. Gender differences on suicide outcomes and problematic cannabis use were assessed using chi-squared analyses and, given that no differences were found on these key variables, gender analyses were not further explored. Logistic-regression analyses were conducted to examine the associations between problematic cannabis use, impulsiveness, and childhood trauma with suicidal thoughts and behaviors. Based on significant logistic-regression results, the mediating role of total impulsiveness and attentional impulsiveness scores on the relationship between problematic cannabis use and suicidal thoughts and behaviors were examined using bootstrapping techniques based on 5000 resamples, to determine 95% confidence limits through model four in PROCESS [49]. Trauma was evaluated as a continuous measure, with low and high points plot generated by the moderation analyses executed by the statistical software. Finally, to examine the moderating role of childhood trauma in the relationship between problematic cannabis use and suicidal thoughts and behaviors, bootstrapping techniques based on 5000 resamples, to determine 95% confidence limits through model one in PROCESS [49], were used. Notably, for the purposes of analyses, all suicide-behaviors variables were coded such that no = 0 and yes = 1.

## 3. Results

### 3.1. Extent of Suicidal Thoughts and Behaviors

Over half of the student sample (52.1%, *n* = 275) reported experiencing suicidal ideation in their lifetime, with one-quarter reporting ideating within the past 12 months (25.9%, *n* = 137). Twelve percent (*n* = 64) of the sample reported a lifetime suicide attempt, and 2.7% (*n* = 14) reported an attempt in the past 12 months. Lifetime and past-12-months suicidal ideation did not differ significantly according to gender, *p* = 0.07 and *p* = 0.37, respectively, and too few males reported suicide attempts to statistically assess gender differences. Specifically, lifetime suicide ideation was reported among 54.0% (*n* = 218) of females and 44.6% (*n* = 54) of males, whereas past-12-months suicidal ideation was reported among 26.5% (*n* = 107) of females and 22.3% (*n* = 27) of males. Suicide attempts were reported by 13.4% (*n* = 54) and 6.6% (*n* = 8) females and males, respectively. Similarly, due to the low number of students reporting suicide attempts in the past 12 months, further analysis of this variable was not conducted. Finally, over one-third of students (35.4%, *n* = 189) reported a current mental health disorder.

### 3.2. Problematic Cannabis Use among University Students

In relation to frequency of cannabis use, 53.5% (*n* = 288) of participants reported no use, whereas 36.0% (*n* = 194) reported non-problematic cannabis use, 3.5% (*n* = 19) reported potential problematic use, and 6.9% (*n* = 37) met the cut-off for possible cannabis-use disorder (CUD). Thus, in the current sample, 10.4% met the cut-off scores for potential problematic cannabis use or possible CUD. Cannabis-use total CUDIT cut-off scores for females (M = 3.08) and males (M = 3.28) did not differ, (χ^2^ (3) = 1.251, *p* = 0.677).

### 3.3. Associations to Problematic Cannabis Use

As shown in Table 1, a series of bivariate correlations revealed that lifetime SI, past-12-months SI, and lifetime SA had moderate positive associations with childhood trauma (*p*’s < 0.01). While total impulsiveness scores had weak positive relations to both lifetime (*p* = 0.01) and past-12-months (*p* < 0.01) SI, attentional impulsiveness demonstrated relationships to lifetime (*p* < 0.01) and past-12-months (*p* < 0.01) SI as well as lifetime SA (*p* = 0.01). Similarly, problematic cannabis use was significantly associated with each of the suicide measures, although these relationships comprised small effect sizes (*p’s* < 0.01, respectively). Problematic cannabis-use scores were also positively, albeit weakly, associated with both total impulsiveness (*p* < 0.01) and attentional impulsiveness (*p* < 0.01) scores. The small correlations observed between cannabis use and suicide ideation and attempts are consistent with early reports in the literature [19,50].

### 3.4. Problematic Cannabis Use as a Predictor of Suicidal Outcomes

Binary logistic regressions were first conducted to investigate whether problematic cannabis use was associated with suicide thoughts and behaviors. As seen in Table 2, problematic cannabis use was found to have a significant relationship with lifetime SI (*p* = 0.003), past 12-months SI (*p* = 0.005), and lifetime SA (*p* = 0.005).

The second set of binary logistic regressions were conducted to examine whether impulsiveness was related to suicidal thoughts and behaviors. When assessing BIS total scores as the sole independent variable, impulsiveness was significantly related to lifetime SI (*p* = 0.001), past-12-months SI (*p* < 0.001), and lifetime SA (*p* = 0.05; Table 3). Next, the three higher-order BIS subscales, attentional impulsiveness, motor impulsiveness, and nonplanning impulsiveness, were added simultaneously into the second model for each suicide outcome. These models were significant for lifetime SI (*p* < 0.001), past-12-months SI (*p* < 0.001), and lifetime SA (*p* = 0.006). When examining the contributions of each predictor, attentional impulsiveness was significantly related to lifetime SI (*p* < 0.001), past-12-months SI (*p* < 0.001), and lifetime SA (*p* = 0.001), whereas motor impulsiveness and nonplanning impulsiveness were not significant in these models. For detailed model statistics, see Table 3.

The final series of binary logistic regressions were performed to examine whether trauma experiences were related to suicidal thoughts and behaviors. Total trauma scores were significant in the case of SI lifetime (*p* < 0.001), past-12-months SI (*p* < 0.001), and lifetime SA (*p* < 0.001; Table 4).

### 3.5. Mediating Role of Impulsiveness in the Relationship between Problematic Cannabis Use and Suicidal Behaviors

**Figure 1:**
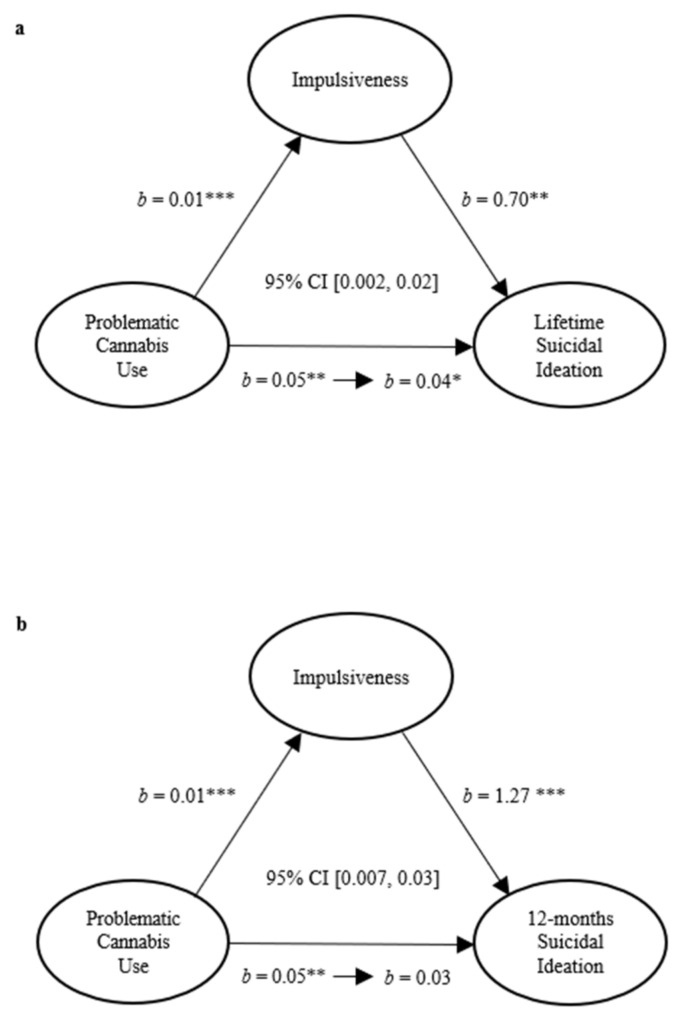
The mediating role of impulsiveness in the relationship between cannabis use and lifetime suicidal ideation (**a**) and past-12-months suicidal ideation (**b**). *** *p* < 0.001, ** *p* < 0.01, * *p* < 0.05.

It was of particular interest to explore the pathways through which impulsiveness may explain the relationship between problematic cannabis use with suicidal thoughts and behaviors. As predicted, impulsiveness mediated the relationship between problematic cannabis use and lifetime SI (*b* = 0.01, 95% CI [0.002, 0.02]), although the direct relationship between problematic cannabis use and lifetime SI remained significant with impulsiveness in the model, *b* = 0.04 and *p* < 0.05, as depicted in Figure 1a. Interestingly, impulsivity also mediated the relation between problematic cannabis use and past-12-months SI (*b* = 0.02, 95% CI [0.007, 0.03]). As shown in Figure 1b, in this instance, once impulsiveness was included in the model, the direct relationship between problematic cannabis use and past-12-months SI was no longer significant, *b* = 0.03 and *p* = 0.051. In contrast to these models, impulsiveness did not mediate the relationship between problematic cannabis use and lifetime SA (*b* = 0.01, 95% CI [−0.004, 0.02]).

It was also of interest to examine whether impulsiveness subscales acted as mediators between problematic cannabis use and indices related to suicide. Attentional impulsiveness was included in these analyses, as it was the only subscale that significantly related to suicidal thoughts and behaviors in the binary logistic regressions. Attentional impulsiveness mediated the relationship between problematic cannabis use and both lifetime SI (*b* = 0.02, 95% CI [0.009, 0.03]), and lifetime SA (*b* = 0.02, 95% CI [0.004, 0.03)]. Moreover, attentional impulsiveness mediated the relationship between problematic cannabis use and past-12-months SI (*b* = 0.02, 95% CI [0.001, 0.04]). For full statistics, see Supplementary Data (Supplemental Table S1).

### 3.6. Moderating Role of Trauma in the Relation between Cannabis Use and Suicide Outcomes

Hierarchical logistic regression was conducted to determine whether childhood trauma moderates the relation between cannabis use and suicidal thoughts and behaviors. While the overall models that examined lifetime and past-12-months SI were significant (χ^2^(3) = 80.70, *p* < 0.0001, *R*^2^ = 0.19, and χ^2^(3) = 60.25, *p* < 0.0001, *R*^2^ = 0.16, respectively), a significant interaction was not present between childhood trauma and cannabis use to predict lifetime SI (*b* = 0.004, *z* = 1.38, *p* = 0.17) or past-12-months SI (*b* = 0.001, *z* = 0.50, *p* = 0.62). When examining lifetime SA, the overall model was significant, χ^2^(3) = 57.02, *p* < 0.0001, and *R*^2^ = 0.20, and there was a main effect of childhood trauma on lifetime SA, *b* = 0.05, *z* = 4.00, and *p* = 0.0001, as well as a significant interaction between childhood trauma and cannabis use, *b* = 0.01, *z* = 2.28, and *p* = 0.023. The simple effects comprising this interaction revealed that the relationship between cannabis use and lifetime SA was significant at high levels of childhood trauma (*p* = 0.004), which was not apparent when considering low levels of childhood trauma (*p* = 0.39).

## 4. Discussion

Cannabis is a widely used psychoactive substance among emerging adults. In 2019, a Canadian national survey reported 53.3% of adolescents ages 16–19 and 69.3% of young adults ages 20–24 reported having used cannabis in their lifetime [51]. In the current study, nearly half of the students (46.5%) reported cannabis use, and 10.4% met the cut-off scores for potential problematic cannabis use or a possible CUD. The prevalence of cannabis use among university students is concerning given recent estimates that 20% of individuals who use cannabis may develop a CUD [52], in addition to the tentative links to suicide behaviors. Given the frequency of cannabis use among university students, the present study investigated the association between cannabis use and suicidal thoughts and behaviors among young adults and further assessed risk factors that supported this relationship. 

Thoughts of suicide were common in the current study, with over half of students reporting suicidal thoughts at some time in their lives and 25% reporting past-12-months SI. Lifetime and past-12-months SI, as well as lifetime SA, were associated with cannabis use, although the effect sizes were small (~2–3%). These findings are consistent with cross-sectional studies showing that individuals with past-30-day cannabis use were more likely to have experienced suicidal ideation and depressive symptoms [53,54]. These relationships may be further moderated by risk factors such as gender, race/ethnicity, and age [29,55]. Of course, in addition to cannabis use, suicidal thoughts and behaviors have also been reported in conjunction with the use of alcohol, anxiolytics/sedatives, and opioids [56], and, in fact, one large longitudinal study reports that the cannabis–suicide ideation link is not explained outside of other substance use [18].

While some studies argue reverse causation, suggesting that the presence of suicidal thoughts can predict later cannabis use, there is evidence for a bidirectional relationship between cannabis use and the depressive symptoms that may precipitate suicide outcomes [57]. The endocannabinoid system has emerged as a key player in the regulation of stress and depression [58,59]. In fact, serum endocannabinoids levels, namely anandamide and N-palmitoiletanolamide, were higher among individuals who attempted suicide in comparison with psychiatric controls [60]. The evolving understanding of the endogenous cannabinoid system, how exogenous cannabinoids may influence mood, and its interaction with stress may help elucidate the cannabis–suicide relationship.

We sought to understand what psychosocial risk factors help explain the relationship between cannabis use and suicidal thoughts and behaviors. Impulsiveness is widely considered a predictor of suicidal thoughts and behaviors and is strongly tied with perceived suicide capability [7]. However, the role of impulsivity in the link between cannabis use and suicide outcomes is uncertain [28]. Thus, we evaluated overall impulsiveness scores and three subdomains of impulsiveness: attentional impulsiveness, motor impulsiveness, and nonplanning impulsiveness. While we hypothesized that impulsivity would consistently mediate the relationship between cannabis use and lifetime and past-12-months SI and lifetime SA, this was not entirely the case, as impulsive traits appeared to selectively mediate the suicide outcomes assessed. Cannabis use was significantly correlated with overall impulsiveness as well as the three impulsiveness subdomains. Moreover, while impulsiveness was significantly associated with lifetime and past-12-months SI, it was not related to SA, and when assessing impulsiveness subscales, attentional impulsiveness, which reflects the inability to focus or complete challenging mental tasks, was the only significant contributor to all three suicide outcomes measured. In contrast, motor and nonplanning impulsiveness, which reflect the tendency to act impulsively, and a lack of consideration for future consequences, respectively, were not significant predictors in these models. Furthermore, overall impulsiveness and attentional impulsiveness mediated the relationship between cannabis use and lifetime SI and past-12-months SI. Together, these findings indicated that impulsive traits, particularly attentional impulsiveness, mediated the cannabis–suicide association, and, thus, should be considered as a risk factor in suicide prediction and prevention among cannabis users.

The interpersonal theory of suicide suggests that complex interactions between risk factors, such as substance use, impulsivity, and childhood trauma, lead to increased levels of suicidality and acquired suicide capability among individuals with impulsive traits [61]. Substance-use disorders, including CUDs, are also more prevalent among individuals with early-life adversity [62,63], and both substance use and childhood trauma are associated with a greater risk of impulsive traits and elevated suicidal thoughts and behaviors [64,65,66,67]. Commensurate with these previous reports, in the present study early-trauma experiences moderated the relationship between cannabis use and lifetime SA, but not for SI outcomes. Specifically, the relationship between cannabis use and suicide attempts was much stronger among individuals who reported high levels of trauma before the age of 18, which is consistent with reports that higher levels of trauma map onto an increased likelihood of problematic cannabis use [60] and a heightened risk of suicide attempts [61].

Several limitations associated with the current study should be considered. Due to the cross-sectional nature of this study, a causal association and direction of the cannabis–suicide link cannot be inferred. The mediation analyses present in this study, therefore, considered the potential explanatory role of impulsivity in the relation between cannabis use and suicide outcomes, however, alternative pathways are possible. It may be the case that cannabis mediates the relationship between impulsivity and suicide. In fact, in testing this alternative model, this was indeed the case. Thus, the directionality cannot be confirmed, as alternative models were also significant. Moreover, while the current study focused on total childhood trauma scores, it would also be interesting to consider individual trauma subtypes experienced before the age of 18, as several studies have observed that different trauma subtypes map on to different suicide outcomes [68,69,70]. While investigating SI and SA in relation to variations in depressive symptoms and trauma subtypes, Bertule et al. observed nuanced predictive qualities of individual subtypes of childhood abuse. Interestingly, emotional abuse may serve as a more distal risk factor, which may set off a cascade of stress-related processes that may increase suicidal ideation. For instance, emotional abuse may increase dissociative traits that are, in turn, linked with depression, which has been identified as a predictor of suicidal ideation [71]. In addition, neglect subtypes may differentially increase risk of a suicide attempt [68,72]. Taken together, prospective studies are needed to elucidate the directional nature of the cannabis–suicide link over time, exploring these additional explanatory variables. Despite having a well-powered study to assess mediation and moderation analyses, the variables being examined, namely suicide attempts, were only apparent in a small proportion of the current sample. Thus, having a larger sample size, or targeted recruitment efforts to increase the proportion of individuals who have experienced suicide behaviors, would have potentially allowed for the examination of more recent (past-12-months) suicide attempts, which we were not able to assess in the current study. There were significantly more females than males, precluding meaningful sex- and gender-based analyses, when considering infrequent outcomes (e.g., suicide behaviors in males). Indeed, sex differences in determinants of suicide risk have been identified [73]. Moreover, sex and gender differences have been reported, wherein cannabis use was a predictor of suicidality in males, whereas suicidality predicted later cannabis use in women [74]. Such findings make it all the more important to evaluate sex- and gender-based factors in the evaluation of the cannabis–suicide link. It might be of significance that in our studies, in general, women were far more likely to volunteer as participants, which may affect the conclusions that can be drawn. Lastly, more detailed information, such as the frequency and timing of suicidal thoughts and behaviors, could have provided better information concerning the links to SI and SA. However, because of ethical considerations in obtaining this information from students, we were constrained by our REB in asking these detailed questions.

## 5. Conclusions

As many countries have shifted toward cannabis legalization, understanding the benefits and harms associated with cannabis use is important. This may be particularly relevant to young and emerging adults, as it was reported that while overall cannabis legalization did not relate to changes in SI and SA rates in the general population, suicide rates have risen approximately 18% among individuals ages 15–24 since legalization [75]. As hypothesized, the current study found a relation between cannabis use and suicide outcomes, and, moreover, identified mediators (i.e., impulsivity) and moderators (i.e., trauma experiences) in the cannabis–suicide link. Thus, the present study contributes to the understanding of the behavioral and environmental factors that might predict suicidal ideation and attempts among young cannabis users, such that impulsive traits and early trauma experiences may heighten vulnerability to these deleterious outcomes among this population. This research could help inform public health education initiatives to increase awareness of potential harms associated with cannabis use among young adults.

## Figures and Tables

**Table 1:** Pearson correlations among problematic cannabis use, suicidal ideation and attempt, childhood trauma, and impulsiveness.

	1	2	3	4	5	6	7	8	9
1. Problematic cannabis use	-								
2. Lifetime SI	0.13 **	-							
3. Past-12-months SI	0.13 **	0.56 **	-						
4. Lifetime SA	0.13 **	0.36 **	0.40 **	-					
5. Childhood trauma	0.11 *	0.35 **	0.34 **	0.33 **	-				
6. Impulsiveness total	0.18 **	0.14 **	0.21 **	0.09	0.16 **	-			
7. Attentional	0.20 **	0.23 **	0.27 **	0.15 **	0.21 **	0.72 **	-		
8. Motor	0.16 **	0.04	0.10 *	0.02	0.11 *	0.79 **	0.37 **	-	
9. Nonplanning	0.09 *	0.06	0.13 **	0.04	0.07	0.82 **	0.36 **	0.49 **	-

*Note*: 1 = cannabis-use disorders identification test-revised; 2 = lifetime suicidal ideation; 3 = past-12-months suicidal ideation; 4 = lifetime suicide attempt; 5 = Childhood Trauma Questionnaire total score; 6 = Barratt Impulsiveness Questionnaire total score and its three higher-order sub-scales 7 = attentional impulsiveness, 8 = motor impulsiveness; and 9 = nonplanning impulsiveness. ** *p*< 0.01. * *p* < 0.05.

**Table 2:** Binary logistic-regression analysis examining problematic cannabis use as a predictor of suicide outcomes.

	*Wald*	*b* (*SE*)	*OR*	95% CI	*p*	*R* ^2^
Lifetime SI	8.62	0.05(0.02)	1.05	1.02, 1.09	0.003	0.02
Past-12-Months SI	8.00	0.05(0.02)	1.05	1.02, 1.08	0.005	0.02
Lifetime SA	8.00	0.05(0.02)	1.06	1.02, 1.10	0.005	0.03

**Table 3:** Binary logistic-regression analysis examining impulsivity as a predictor of suicidal behaviors.

	*Wald*	*b* (*SE*)	*OR*	95% CI	*p*	*R* ^2^
**Lifetime SI**						
Impulsiveness	10.25	0.81 (0.25)	2.26	1.37, 3.71	0.001	0.03
Attentional	25.69	0.98 (0.19)	2.67	1.82, 3.90	<0.001	
Motor	0.67	−0.21 (0.26)	0.81	0.48, 1.35	0.41	0.07
Nonplanning	0.02	−0.03 (0.23)	0.97	0.62, 1.52	0.90	
**Past-12-Months SI**						
Impulsiveness	22.11	1.36 (0.29)	3.90	2.21, 6.89	<0.001	0.06
Attentional	29.14	1.16 (0.22)	3.20	2.10, 4.88	<0.001	
Motor	0.11	−0.10 (0.29)	0.91	0.51, 1.61	0.74	0.11
Nonplanning	0.73	0.22 (0.26)	1.25	0.75, 2.09	0.39	
**Lifetime SA**						
Impulsiveness	3.80	0.72 (0.37)	2.05	1.00, 4.23	0.05	0.01
Attentional	11.52	0.92 (0.27)	2.50	1.47, 4.24	0.001	
Motor	0.54	−0.28 (0.39)	0.75	0.35, 1.61	0.46	0.04
Nonplanning	0.001	0.01 (0.34)	1.01	0.52, 1.96	0.98	

*Note.* Lifetime SI = lifetime suicidal ideation; Past-12-Months SI = past-12-months suicidal ideation; Lifetime SA = lifetime suicide attempt; Impulsiveness = BIS—total score; Attentional = BIS subscale attentional impulsiveness; Motor = BIS subscale motor impulsiveness; Nonplanning = BIS subscale nonplanning impulsiveness; CI = confidence interval.

**Table 4:** Binary logistic-regression analysis examining childhood trauma as a predictor of suicidal behaviors.

	*Wald*	*b* (*SE)*	*OR*	95% CI	*p*	*R* ^2^
**Childhood trauma**						
Lifetime SI	54.05	0.08 (0.01)	1.08	1.06, 1.10	<0.001	0.18
Past-12-Months SI	48.27	0.06 (0.01)	1.06	1.04, 1.08	<0.001	0.15
Lifetime SA	43.31	0.06 (0.01)	1.07	1.05, 1.08	<0.001	0.16

*Note.* Lifetime SI = lifetime suicidal ideation; Past-12-Months SI = past-12-months suicidal ideation; Lifetime SA = lifetime suicide attempt; Childhood Trauma = CTQ total score; CI = confidence interval.

## Data Availability

These data were not approved by the REB to be made publicly available.

## Supplementary Materials

The following supporting information can be downloaded at: https://www.mdpi.com/article/10.3390/ijerph19159307/s1. Table S1. The mediating role of impulsiveness on problematic cannabis use and suicidal thoughts and behaviors. Figure S1: Hypothesis #1: Problematic cannabis use would be associated with increased reports of suicidal ideation and suicide attempt; Figure S2: Hypothesis #2: Impulsive traits, including the three subdomains considered, would mediate the relationship between higher problematic cannabis use and greater endorsement of suicidal thoughts and behaviors; Figure S3: Hypothesis #3: Trauma experiences before the age of 18 would moderate the relationship between cannabis use and all suicide outcomes assessed, such that heavier cannabis use would predict greater endorsements of suicide ideation and attempts, especially among those with higher trauma scores.
