# Supplementary material for: Examining Risk Factors in the Cannabis–Suicide Link: Considering Trauma and Impulsivity among University Students
Source: Int J Environ Res Public Health. 2022 Jul 29;19(15):9307. doi: 10.3390/ijerph19159307 (PMC9368410; doi:10.3390/ijerph19159307)
Supplement: Supplementary file 1 [file ijerph-19-09307-s001.zip › ijerph-1809964-supplementary.pdf]

Examining Risk Factors in the Cannabis–Suicide Link: Considering Trauma and Impulsivity among University Students

Supplementary Data

Table S1. The mediating role of impulsiveness on problematic cannabis use and suicidal thoughts and behaviors.

	b	*SE*	*p*	95% CI
**BIS Total Score and Lifetime SI**				
a path	0.01	0.003	< .001	0.01, 0.02
b path	0.70	0.26	.007	0.19, 1.20
c path	0.05	0.02	.003	1.02, 1.09
c′ path	0.04	0.02	.016	0.01, 0.08
**BIS Total Score and Past 12-Months SI**				
a path	0.01	0.003	< .001	0.01, 0.02
b path	1.27	0.29	<.001	0.69, 1.84
c path	0.05	0.02	.005	1.02, 1.08
c′ path	0.03	0.02	.051	-0.0001, 0.68
**BIS Total Score and Lifetime SA**				
a path	0.01	0.003	< .001	0.01, 0.02
b path	0.54	0.38	.15	-2.05, 1.29
c path	0.06	0.02	.003	1.02, 1.10
c′ path	0.05	0.02	.01	0.01, 0.09
**Attentional Impulsiveness and Lifetime SI**				
a path	0.02	0.004	< .001	0.01, 0.03
b path	0.85	0.18	< .001	0.50, 1.20
c path	0.05	0.02	.003	1.02, 1.09
c′ path	0.04	0.02	.044	0.001, 0.07
**Attentional Impulsiveness and Past 12-Months SI**				
a path	0.02	0.004	< .001	0.01, 0.03
b path	1.15	0.20	< .001	0.75, 1.54
c path	0.05	0.02	.005	1.02, 1.08
c′ path	0.03	0.02	.10	-0.01, 0.06
**Attentional Impulsiveness and Lifetime SA**				
a path	0.02	0.004	< .001	0.01, 0.03
b path	0.75	0.26	.003	0.25, 1.25
c path	0.06	0.02	.003	1.02, 1.10

c' path	0.05	0.02	.023	0.01, 0.09

Note. BIS = Barratt Impulsiveness Scale – 11; SI Lifetime = lifetime suicidal ideation; SI 12-Months = past 12-months suicidal ideation; SA Lifetime = lifetime suicide attempt.

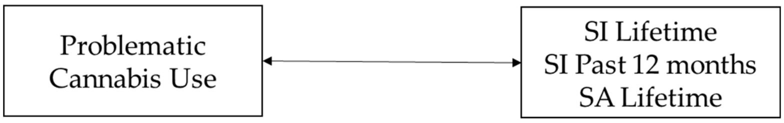

Figure S1. Hypothesis #1: Problematic cannabis use would be associated with increased reports of suicidal ideation and suicide attempt.

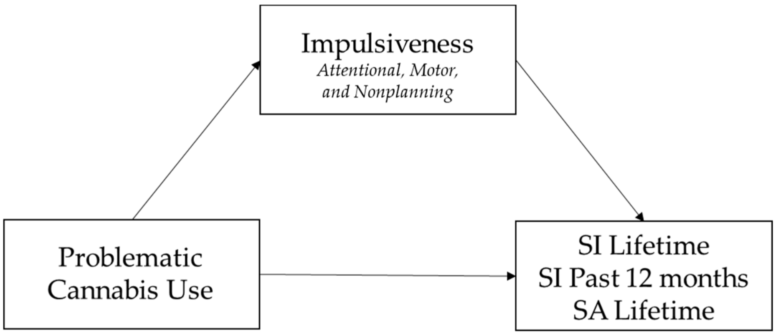

Figure S2: Hypothesis #2: Impulsive traits, including the three subdomains considered, would mediate the relationship between higher problematic cannabis use and greater endorsement of suicidal thoughts and behaviors.

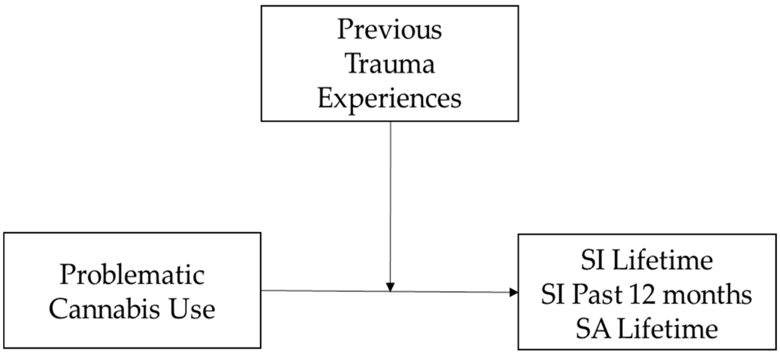

Figure S3. Hypothesis #3: Trauma experiences before the age of 18 would moderate the relationship between cannabis use and all suicide outcomes assessed, such that heavier cannabis use would predict greater endorsements of suicide ideation and attempts, especially among those with higher trauma scores.
